# A Lattice Boltzmann Thermal Model for Predicting Melt Pool Geometry in Selective Laser Melting of AlSi10Mg and 316L Stainless Steel

**DOI:** 10.3390/ma19071297

**Published:** 2026-03-25

**Authors:** Rigoberto Guzmán-Nogales, Luis A. Reyes-Osorio, Guadalupe M. Hernández-Muñoz, Alex Elías-Zúñiga, Omar E. López-Botello, Carlos Garza-Rodríguez, Patricia C. Zambrano-Robledo

**Affiliations:** 1 Centro de Innovación e Investigación en Ingeniería Aeronáutica, Facultad de Ingeniería Mecánica y Eléctrica, Universidad Autónoma de Nuevo León, San Nicolás de los Garza 66455, N.L., Mexico; rigo.guzmann@tec.mx (R.G.-N.); luis.reyessr@uanl.edu.mx (L.A.R.-O.); guadalupe.hernandezmn@uanl.edu.mx (G.M.H.-M.); carlos.garzarg@uanl.edu.mx (C.G.-R.); 2 Tecnologico de Monterrey, Institute of Advanced Materials for Sustainable Manufacturing, Ave. Eugenio Garza Sada 2501, Col. Tecnológico, Monterrey 64700, N.L., Mexico; aelias@tec.mx; 3 Laboratorio Nacional de Manufactura Aditiva y Digital MADIT, Autopista al Aeropuerto Internacional Mariano Escobedo, Apodaca 66629, N.L., Mexico

**Keywords:** selective laser melting process, lattice boltzmann method, AlSi10Mg, 316L stainless steel

## Abstract

Selective laser melting (SLM) is a complex additive manufacturing process involving rapid laser–material interaction, steep thermal gradients, and phase change phenomena. In this work, a two-dimensional thermal model based on the lattice Boltzmann method (LBM) is developed to simulate the SLM process of AlSi10Mg and 316L stainless steel (316L SS) alloys. The model captures the laser–material interaction, layer-by-layer deposition, phase change behavior, and heat transfer mechanisms, including conduction and convection. Experimental observations of melt pool width and depth were also performed on the microstructures of the two SLM alloys in order to compare the results with the numerical predictions. For the AlSi10Mg alloy, good agreement is obtained, with relative errors of 19.13% in melt pool width and 7.58% in depth, accurately capturing melt pool penetration and remelting behavior. In contrast, moderate deviations are observed for 316L SS, indicating a higher sensitivity to thermophysical properties and suggesting that further model refinement is required. Overall, the results demonstrate the capability of the LBM framework as an efficient and robust tool for analyzing thermal behavior in SLM and for supporting process parameter optimization.

## 1. Introduction

Selective laser melting (SLM) is a powerful methodology that involves heating and melting metallic powder alloys (e.g., nickel, titanium, aluminum, steel-based, etc.) for building three-dimensional components in a layer-by-layer manner. The process is complex because it uses a high-power laser as an energy source to scan successive thin powder layers and then generate a small volume of molten material known as a melt pool. Generally, the melt pool geometry displays a semicircular shape with a width and depth (melt pool dimensions). The complete macrostructure of an SLM material comes when several melt pools, small circular or cylindrical parts [1], form a relatively tidy path of segments during the melting process [2]. The SLM processing parameters that strongly influence the geometry of the melt pool are laser power, scanning speed, hatch spacing between consecutive tracks, melting strategy, layer thickness, and spot irradiation time [3]. Therefore, an appropriate melt pool geometry significantly influences a successful SLM process.

Several experimental investigations have been conducted to characterize melt pool geometry and its dependence on processing parameters in laser melting of AlSi10Mg and 316L SS alloys. Hofmann et al. [4] analyzed 677 single tracks produced by laser powder bed fusion (LPBF) of 316L SS, considering three different layer thicknesses and four distinct laser spot sizes. For each combination of layer thickness and spot size, the laser power and scanning speed were systematically varied, generating an extensive experimental dataset of melt pool geometries. The primary objective of this work was to establish a comprehensive technical database describing melt pool width and depth under controlled processing conditions. Based on these measurements, multilinear regression models were developed to predict melt pool dimensions and to construct process maps quantifying the influence of processing parameters on melt pool shape and instability. The study demonstrated that melt pool geometry acts as a critical intermediate variable linking processing parameters with final material properties, such as mechanical strength, porosity formation, and microstructure evolution in additively manufactured parts. Zhao et al. [5] investigated the microstructure of SLM 316L SS fabricated by a multi-laser strategy, distinguishing between the single laser forming zone and the overlap zone. In the single laser forming zone, the melt pools exhibited a relatively irregular morphology, with average width and depth values of approximately 190.35 μm and 81.44 μm, respectively. In contrast, the overlap zone presented a more uniform and semi-elliptical melt pool structure, with increased average dimensions of about 200.01 μm in width and 120.22 μm in depth. The authors attributed this difference to thermal accumulation and secondary remelting effects in the overlap region, which extended the melt pool lifetime and promoted deeper penetration. Xu et al. [6] developed a three-dimensional finite element model to analyze the thermodynamic behavior of the melt pool during SLM processing of AlSi10Mg. The model was validated through single-track experiments, showing good agreement between simulated and measured melt pool dimensions. Under processing conditions of 250 W laser power and a scanning speed of 1000 mm/s, the experimentally measured melt pool exhibited an average width of approximately 143 μm and a depth close to 53 μm. Yue et al. [7] further investigated the effect of remelting strategies on melt pool formation using a numerical–experimental framework. Their results indicated that the remelting strategy produces a more symmetric and compact melt pool morphology compared with the conventional SLM process, which contributes to a reduction in porosity and promotes the formation of finer microstructures, leading to improved mechanical properties and hardness. In addition, Wang et al. [8] proposed a thermo-structural coupled finite element model to study the thermal evolution and residual stress formation during SLM processing of AlSi10Mg. The numerical predictions were validated with experimental measurements of melt pool geometry and residual stresses. Their results demonstrated that increasing laser power leads to higher peak temperatures, longer melt pool lifetimes, and enhanced fluidity of the molten material, which ultimately results in larger melt pool dimensions.

Therefore, for a better understanding of the SLM process, predictive thermal models are essential to properly describe the physical phenomena involved, in particular the melt pool formation during the complex interaction between the powder bed and the laser beam. Among these numerical approaches, Körner et al. [9] followed a mesoscopic method to simulate the melting and re-solidification of a selective electron beam melting (SEBM) titanium alloy. The developed approach based on the lattice Boltzmann method (LBM) can predict several conditions, such as the stochastic effect, capillary and wetting effect, balling effect, etc., which strongly influence the consolidation of the melt pool. Lopez et al. [10] developed a coupled cellular automata–finite element (CA-FE) model for the prediction of the microstructure of an SLM aluminum alloy. The model is able to predict the dimensions of the melt pool, as well as the microstructure evolution. Li et al. [11] also used the finite element method (FEM) to simulate the temperature distribution during the laser melting process. The numerical results led to an optimal value in the melt pool width and depth for a successful SLM process at a fixed laser power and scan speed. Consequently, Liu et al. [12] developed another important SLM model through FEM. In that approach, a three-dimensional transient model was established to calculate the dimensions of the melt pool and the thermal variables of the SLM AlSi10Mg alloy. These FEM-based models with outstanding results are widely reported in the literature, as reviewed in [13]; however, a limited number of studies have employed homogeneous mesoscopic approaches to simulate the physical phenomena involved in the SLM process. For example, Körner et al. [9] developed powerful methods of SLM using the LBM. In Rai et al.’s [14] study, the LBM formulation was implemented to simulate the melt pool dynamics and grain growth of the SLM IN718 superalloy. Zheng et al. [15] utilized the LBM to study the melt pool dynamics and the resultant porosity during the manufacture of Inconel 625 via SLM. Several instabilities inside the melt pool, such as lack of fusion, trapped gas, etc., were associated with different processing parameters and powder packing densities. Dai et al. [16] formulated a lattice Boltzmann model showing that fast scanning speeds promote the formation of partially melted powder particles. Therefore, in this research, a two-dimensional thermal model based on the lattice Boltzmann method (LBM) is developed to predict the temperature evolution and melt pool geometry of AlSi10Mg and 316L stainless steel alloys processed by SLM. The proposed model serves as a fast optimization-oriented tool, providing preliminary insight into the heat transfer mechanisms governing the SLM process while accounting for the most relevant processing parameters. To validate the proposed numerical model, SLM-fabricated 316L SS and AlSi10Mg samples were produced and experimentally characterized. The width and depth of multiple melt pools were measured from the optical microstructures of both alloys.

The main novelty of the present work lies in the development and validation of a simplified lattice Boltzmann thermal model capable of predicting melt pool geometry for two different metallic alloys processed by selective laser melting. Unlike several previous studies that focus on a single material system or rely exclusively on numerical simulations, the present approach combines the LBM formulation with experimental characterization of melt pool dimensions obtained from metallographic observations of AlSi10Mg and 316L SS samples.

Furthermore, the proposed model is designed as a computationally efficient framework that captures the dominant thermal mechanisms governing melt pool formation while maintaining a relatively low computational cost compared with fully three-dimensional multiphysics models. This allows the model to be used as a practical tool for preliminary analysis of process parameters and melt pool behavior in selective laser melting.

## 2. Materials and Methods

### 2.1. Experimental Setup

An SLM 280HL system (SLM Solutions Inc., Lübeck, Germany) equipped with a 400 W continuous-wave ytterbium fiber laser was used to manufacture all samples (AlSi10Mg and 316L SS). The laser is water-cooled, operates in continuous mode, has a spot diameter of approximately 70 μm, and emits radiation at a wavelength of 1060 nm. The SLM machine employs an argon-filled chamber to ensure an inert processing atmosphere and provides a working volume of 280 mm × 280 mm × 350 mm for part fabrication.

During the SLM process, a homogeneous layer of metallic powder is deposited onto the build plate using a recoater blade and subsequently scanned by the laser beam. The volumetric energy density (*E*) applied to the powder bed is defined as

(1) $$E=\frac{P}{v\times HS\times Lt}$$

where *P* denotes the laser power (W), *v* is the scanning speed (mm/s), HS represents the hatch spacing (mm), defined as the perpendicular distance between adjacent scan tracks, and Lt is the powder layer thickness (mm).

### 2.2. SLM Samples Fabrication

**Figure 1:**
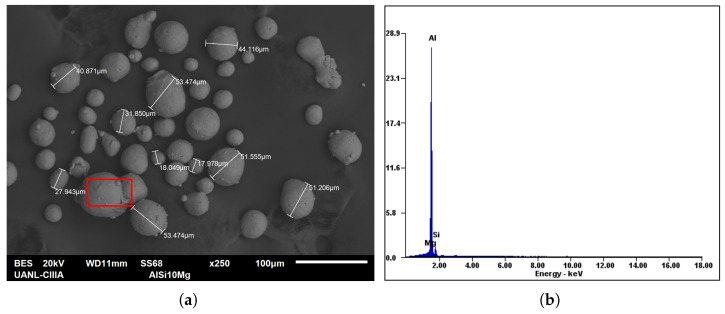
SEM image of the AlSi10Mg metal powder: (**a**) Size of some particles. (**b**) EDS analysis performed on the region indicated by the red box in (**a**).

The AlSi10Mg alloy in its nearly spherical shape (20–63 μm), shown in Figure 1 and supplied by LPW Technology, was selected to fabricate cubic parts of 10 mm × 10 mm × 10 mm using the SLM process. The particle size and chemical composition (wt. %) were analyzed by Scanning Electron Microscopy (SEM) and Energy Dispersive Spectroscopy (EDS), respectively. The EDS observation was carried out on one of the powder particles to reveal its chemical structure and major alloying elements. The spectrum reveals that aluminum is the dominant element with a measured content of approximately 87.6 wt.%, followed by silicon with 11.30 wt.% and magnesium with 1.10 wt.%, as shown in Table 1. In addition to the elemental composition of the material in its spherical phase (powder), the chemical composition of the solid phase (i.e., as an SLM sample) was obtained by X-ray Fluorescence Spectroscopy (XRF). Although the XRF analysis does not provide a complete chemical composition as given by the manufacturer, it presents the major alloying elements similarly to the EDS results.

The manufacturing of the samples was performed with a 30 μm layer thickness, 100 W laser power, 100 mm/s scanning speed, and 100 μm hatch spacing under a controlled atmosphere with argon gas. The generated inert ambiance allowed controlling the oxygen levels to less than 0.1% during the SLM process. The manufacturing process started with the preheating of the substrate in order to reduce internal forces and deformation, which are some of the main factors that affect the final properties of the material [17]. Therefore, the building platform was preheated at 200 °C.

**Table 1:** Elemental composition (wt. %) of the AlSi10Mg metallic powder and SLM sample [18].

Element	Fe	Mg	Ni	Si	Zn	Al
Powder	–	1.10	–	11.30	–	Bal.
SLM sample	0.17	1.09	0.006	10.82	0.0067	Bal.

In the SLM process, laser energy absorptivity is another key parameter governing the interaction between the laser beam and the material. This intrinsic property, which varies among metallic materials [19], determines the effective fraction of laser energy absorbed by the powder bed. Although the absorptivity factor is commonly assumed to be constant in numerical simulations [20], it is known to depend on several material and processing parameters, among which the laser wavelength, mean particle size, and particle morphology are the most influential [21]. For the AlSi10Mg alloy, a laser energy absorptivity value of 0.09 is adopted in the present numerical simulations [11]. In addition, the ambient temperature of the building chamber is assumed to be 25 °C.

**Figure 2:**
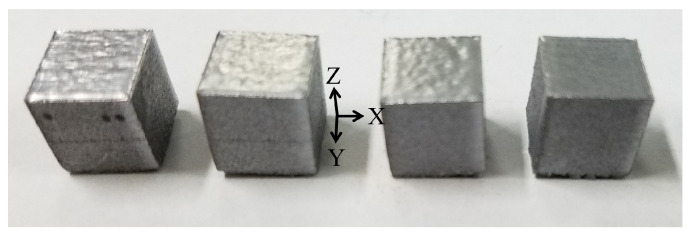
SLM AlSi10Mg parts [18].

Figure 2 shows the AlSi10Mg specimens fabricated by SLM. The samples were cubic in shape with dimensions of 10 mm × 10 mm × 10 mm, half-sectioned in two planes (XZ and YZ), and analyzed to examine their microstructure.

**Figure 3:**
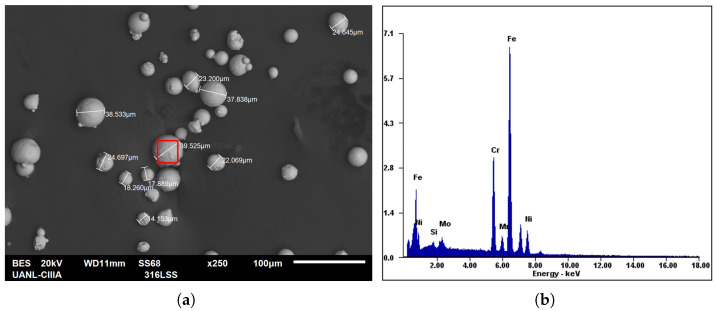
SEM image of the 316L SS metal powder: (**a**) size of some particles and (**b**) EDS analysis performed on the region indicated by the red box in (**a**).

For the 316L SS alloy, nearly spherical powder particles with a size range of 10–45 μm were used, as shown in Figure 3. The powder was supplied by LPW Technology and was employed to fabricate cubic samples with dimensions of 10 mm × 10 mm × 10 mm. The particle size and chemical composition (wt. %) of the powder were analyzed by Scanning Electron Microscopy (SEM) and Energy Dispersive Spectroscopy (EDS), respectively. The quantitative EDS analysis shows that iron is the dominant element with a measured concentration of approximately 64.87 wt.%, followed by chromium (18.38 wt.%), nickel (11.65 wt.%), molybdenum (2.48 wt.%), manganese (1.61 wt.%), and silicon (1.00 wt.%), as shown in Table 2. This chemical composition was obtained from local EDS measurements performed on representative powder particles. Then, once the SLM samples were manufactured, their chemical composition was also analyzed by X-ray Fluorescence Spectroscopy (XRF), showing the major alloying elements. The small differences between the measured values reported in Table 2 arise from the different spatial scales of the analytical techniques employed; while EDS measurements provide localized compositional information from a small analyzed area, XRF represents the average chemical composition over a larger material volume. In addition, the rapid melting and solidification associated with the SLM process may promote local microsegregation effects, which can also contribute to slight variations in the measured elemental concentrations.

The SLM process was carried out using the SLM 280HL machine, with the following processing parameters: 30 μm layer thickness, 400 W laser power, 110 μm hatch spacing, and 230 mm/s scanning speed. The building chamber was filled with argon gas to maintain oxygen concentrations below 0.1% and prevent part oxidation.

As shown in Figure 3, measurements were performed on several powder particles to verify the particle size distribution provided by the manufacturer (LPW Technology). In addition, an energy-dispersive spectroscopy (EDS) analysis was conducted on a representative particle to determine its elemental composition and identify the major alloying elements.

**Figure 4:**
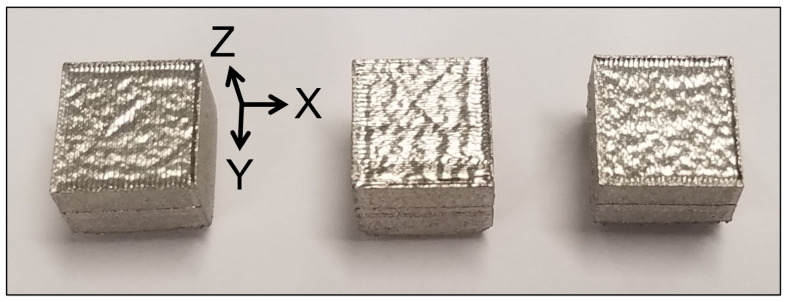
SLM 316L SS parts [18].

Figure 4 shows the 316L SS samples fabricated by SLM. Such specimens were cubic in shape with dimensions of 10 mm × 10 mm × 10 mm, which were also half-sectioned and analyzed to examine their microstructure.

To summarize the SLM processing parameters and thermo-physical properties used in this study, Table 3 lists the corresponding values for both AlSi10Mg and 316L stainless steel alloys. These parameters were used to fabricate the metallic samples with dimensions of 10 mm × 10 mm × 10 mm and to define the thermo-physical properties employed in the numerical simulations.

### 2.3. Experimental Characterization

For the observation of the melt pool morphology in the SLM AlSi10Mg alloy, the samples were etched with Keller’s reagent (190 mL distilled water, 5 mL HNO_3_, 3 mL HCl, 2 mL HF for 10 s). A Carl Zeiss inverted optical microscope was used to examine and measure the melt pool shape. An average of eighty-five melt pool measurements per sample was performed at different locations. The final average size of melt pool was then determined for each sample. Additionally, the grain structure of the SLM AlSi10Mg material was analyzed. For this purpose, the specimens were etched with Keller’s reagent, followed by a second etching step using an electrolytic solution with Barker’s reagent (2.5 mL HBF_4_, 200 mL distilled water during 5 s at 2 V). To observe the microstructure, polarized light was used in the optical microscope.

For the observation of the melt pool morphology in the SLM 316L SS alloy, the mounted and polished samples were etched with Glyceregia (15 mL HCl, 10 mL Glycerol, and 5 mL HNO_3_). The Carl Zeiss inverted optical microscope was then used to observe and measure the melt pool shape. In this case, an average of sixty-five melt pool measurements per sample was obtained at different locations. The final average melt pool size was determined for each sample.

The melt pool dimensions were determined from the acquired optical micrographs using a geometrical fitting procedure. Each melt pool cross-section was approximated by a semi-elliptical profile, which is consistent with the typical morphology observed in single-track SLM processes. The melt pool width was defined as the maximum horizontal distance across the fusion boundary, while the melt pool depth was measured as the maximum vertical distance from the top surface to the deepest point of penetration. Dimensional measurements were obtained by calibrating the micrographs using the scale bar provided in each optical image. A proportional scaling procedure was applied to convert pixel-based distances into micrometer values. The measured width and depth values were recorded individually for each melt pool and subsequently averaged to obtain the representative melt pool dimensions for each processing condition.

## 3. Thermal Modeling

### 3.1. Computational Domain

**Figure 5:**
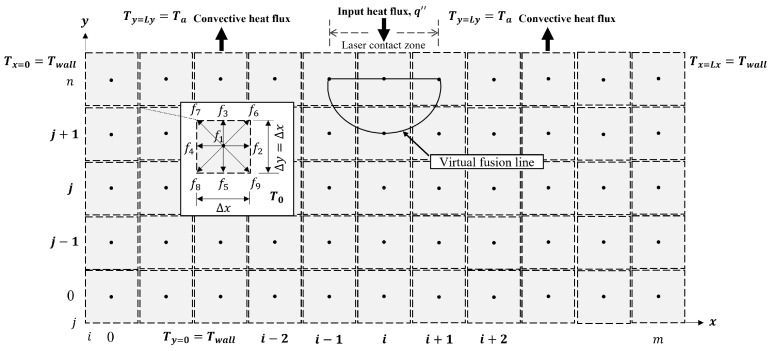
Two-dimensional scheme of SLM via LBM.

The lattice Boltzmann formulation is implemented by adopting the standard D2Q9 lattice structure to define a two-dimensional simulation domain, as illustrated in Figure 5. The computational domain has a length of $1\times 10^{-3}\;m$ and a height of $0.56\times 10^{-3}\;m$. The domain height ($L_{y}$) consists of a $0.5\times 10^{-3}\;m$ substrate thickness together with two deposited powder layers representing the powder bed. In the present study, the substrate is assumed to have the same thermo-physical properties as the powder bed. The domain is discretized into a uniform lattice of 200×112 cells. Each lattice cell contains particle distribution functions whose temporal evolution governs the thermal response of the system. These distribution functions, derived from the lattice Boltzmann equation, are employed to predict the temperature distribution induced by the laser–material interaction within the proposed rectangular domain.

It should be noted that the present numerical model adopts a two-dimensional formulation to represent the thermal behavior during the SLM process. Although the real laser powder bed fusion process is inherently three-dimensional, the 2D approximation allows capturing the dominant heat transfer mechanisms governing melt pool formation while significantly reducing the computational cost. In particular, the 2D model reproduces the main characteristics of the melt pool cross-section, including its width, penetration depth, and the thermal gradients developed beneath the laser interaction zone. These features are directly comparable with the experimental melt pool measurements obtained from metallographic cross-sections.

Nevertheless, some three-dimensional effects, such as melt pool fluid flow, recoil pressure dynamics caused by metal evaporation, and spatial variations along the scanning direction, are not explicitly represented in the present formulation. Therefore, the proposed model should be interpreted as a simplified thermal framework aimed at providing a fast and reliable prediction of melt pool geometry and temperature distribution. Future work may extend the present formulation toward fully three-dimensional simulations in order to capture additional physical mechanisms involved in the SLM process.

From a physical standpoint, the present numerical formulation focuses primarily on the thermal aspects of the SLM process. The dominant heat transfer mechanisms considered in the simulation include laser energy absorption, heat conduction within the material, and convective heat exchange with the surrounding environment. These mechanisms are sufficient to capture the principal temperature evolution and melt pool geometry in the cross-section of the processed material.

In contrast, several complex physical phenomena associated with melt pool dynamics are not explicitly included in the present model. In particular, fluid flow driven by Marangoni convection, recoil pressure induced by metal evaporation, and mass loss due to evaporation are not considered. These effects may influence the detailed shape and stability of the melt pool, especially under high-laser-power conditions where strong surface tension gradients and vaporization can occur.

The omission of these mechanisms may lead to moderate deviations in the predicted melt pool dimensions, particularly in terms of melt pool depth and flow-induced asymmetries. Nevertheless, the simplified thermal formulation adopted in this work still provides a reliable first-order prediction of melt pool geometry and temperature distribution. Such simplified approaches are commonly used in thermal modeling of SLM processes to provide computationally efficient insights into the influence of processing parameters on melt pool formation.

### 3.2. Lattice Boltzmann Formulation

The general kinetic equation to describe the propagation of information in a representative scheme via LBM can be written as [20]

(2) $$\frac{\partial f_{k}\left(\vec{r},t\right)}{\partial t}+{\vec{c}}_{k}\cdot \nabla f_{k}\left(\vec{r},t\right)=\Omega_{k}$$

where $\Omega_{k}$ is a collision operator, which represents the rate of local change between the final and initial state of the distribution function $f_{k}$ due to the collision process [27]. Based on the Bhatnagar–Gross–Krook (BGK) collision scheme, $\Omega_{k}$ is given by [20,28].

(3) $$\Omega_{k}=-\frac{1}{\tau }[f_{k}\left(\vec{r},t\right)-f_{k}^{eq}\left(\vec{r},t\right)]$$

By substituting Equation (3) into Equation (2), and then discretizing, it yields the discretized lattice Boltzmann Equation [20]

(4) $$f_{k}\left(\vec{r}+{\vec{c}}_{k}\Delta t,t+\Delta t\right)=f_{k}\left(\vec{r},t\right)+\frac{\Delta t}{\tau }\left[f_{k}^{eq}\left(\vec{r},t\right)-f_{k}\left(\vec{r},t\right)\right]$$

where $f_{k}$ is the particle distribution function at the lattice position $\vec{r}$ and at time *t*, moving with a microscopic velocity ${\vec{c}}_{k}$ in the *k*-direction along the lattice link. τ is a relaxation factor related to collision frequency ω as τ=Δt/ω, and $f_{k}^{eq}$ is the equilibrium particle distribution function known as the Maxwell–Boltzmann equilibrium distribution function.

The relaxation time is related to the thermal diffusivity, the velocity *c*, and the time step in the lattice Boltzmann formulation by [28].

(5) $$\tau =\frac{3\alpha }{c^{2}}+\frac{\Delta t}{2}$$

For the D2Q9 lattice structure of this methodology, the lattice Boltzmann equation for the temperature distribution function into the simulation domain is given by [20].

(6) $$f_{k}\left(x+\Delta x,y+\Delta y,t+\Delta t\right)=f_{k}\left(x,y,t\right)+\frac{\Delta t}{\tau }\left[f_{k}^{eq}\left(x,y,t\right)-f_{k}\left(x,y,t\right)\right]$$

where k=1,2,…,9 represents the nine velocities ${\vec{c}}_{k}$ of the selected lattice structure.

The velocity vectors for the propagation of information (temperature distribution) along the corresponding lattice links are ${\vec{c}}_{1}=\left(0,0\right)$, ${\vec{c}}_{2}=\left(c,0\right)$, ${\vec{c}}_{3}=\left(0,c\right)$, ${\vec{c}}_{4}=\left(-c,0\right)$, ${\vec{c}}_{5}=\left(0,-c\right)$, ${\vec{c}}_{6}=\left(c,c\right)$, ${\vec{c}}_{7}=\left(-c,c\right)$, ${\vec{c}}_{8}=\left(-c,-c\right)$, and ${\vec{c}}_{9}=\left(c,-c\right)$, where c=Δx/Δt=Δy/Δt. Δx and Δt are the lattice space and the lattice time step, which equal unity for this lattice Boltzmann formulation.

Consequently, the scalar factor to characterize the diffusion problem can be deduced from the distribution function $f_{k}$ in the following way:

(7) $$T\left(x,y,t\right)=\sum_{k=1}^{9}f_{k}\left(x,y,t\right)$$

The equilibrium distribution function $f_{k}^{eq}$ can be obtained as follows:

(8) $$f_{k}^{eq}\left(x,y,t\right)=w_{k}T\left(x,y,t\right)$$

$w_{k}$ stands for the weight factors of the corresponding distribution functions. These factors must satisfy the relation $\sum_{k=1}^{9}w_{k}=1$ and are defined in the following way:

(9) $$\begin{array}{ccc} w_{k}=\frac{4}{9} & \; & \mathrm{for}\;k=1 \end{array}$$

(10) $$\begin{array}{ccc} \;w_{k}=\frac{1}{9} & \; & \mathrm{for}\;k=2,3,4,5 \end{array}$$

(11) $$\begin{array}{ccc} \;w_{k}=\frac{1}{36} & \; & \mathrm{for}\;k=6,7,8,9 \end{array}$$

From Equation (8) and due to the system being at the initial temperature $T_{0}$ (preheating temperature of the laser melting system), it is possible to start the calculations with the relation given by

(12) $$f_{k}^{eq}\left(x,y,0\right)=w_{k}T_{0}\left(x,y,0\right)$$

Once the initial condition has been established by using Equation (12), the solution of Equation (6) for the evolution of the system is performed by two general steps known as collision and streaming, which can be written in the following way:

collision:

(13) $$f_{k}\left(x,y,t+\Delta t\right)=f_{k}\left(x,y,t\right)+\frac{\Delta t}{\tau }[f_{k}^{eq}\left(x,y,t\right)-f_{k}\left(x,y,t\right)],\;k=1,2,\ldots ,9$$

streaming:

(14) $$f_{k}\left(x+\Delta x,y+\Delta y,t+\Delta t\right)=f_{k}\left(x,y,t+\Delta t\right),\;k=1,2,\ldots ,9$$

Another important point for the beginning of the calculations is that the equilibrium distribution function $f_{k}^{eq}$ in Equation (8) can be related to the temperature distribution function as

(15) $$\sum_{k=1}^{9}f_{k}^{eq}\left(x,y,t\right)=\sum_{k=1}^{9}w_{k}T\left(x,y,t\right)=T\left(x,y,t\right)$$

### 3.3. Phase-Change Formulation

In order to consider the phase change during the simulation process, the enthalpy equation is coupled with the lattice Boltzmann formulation, which can be written as [16]

(16) $$f_{k}\left(x+\Delta x,y+\Delta y,t+\Delta t\right)=f_{k}\left(x,y,t\right)+\frac{\Delta t}{\tau }\left[f_{k}^{eq}\left(x,y,t\right)-f_{k}\left(x,y,t\right)\right]-w_{k}\frac{L}{C_{p}}\left(f_{l}^{t}-f_{l}^{t-\Delta t}\right)$$

where *L* is the latent heat related to the total enthalpy at each time step. The enthalpy can be obtained from

(17) $$H=C_{p}T+Lf_{l}$$

where $f_{l}$ is the liquid fraction and can be formulated as

(18) $$f_{l}=\left\{\begin{array}{cc} 0 & H<H_{s}\\ \frac{H-H_{s}}{H_{l}-H_{s}} & H_{s}\leq H\leq H_{l}\\ 1 & H>H_{l} \end{array}\right.$$

where $H_{s}$ and $H_{l}$ are the values for the enthalpy at the solidus and liquidus temperature, respectively.

The last term in Equation (16) represents the latent heat source associated with the solid–liquid phase transition. Physically, this term accounts for the absorption or release of latent heat during melting and solidification processes. The quantity $\left(f_{l}^{t}-f_{l}^{t-\Delta t}\right)$ represents the temporal variation of the liquid fraction between two consecutive time steps, which determines the amount of latent heat involved in the phase transition.

From a numerical standpoint, the phase change is implemented using an enthalpy-based formulation. At each time step, the temperature field obtained from the lattice Boltzmann equation is used to compute the total enthalpy according to Equation (17). Subsequently, the liquid fraction $f_{l}$ is updated using the piecewise relation given in Equation (18), depending on whether the local enthalpy lies within the solid, mushy, or liquid region. The variation in the liquid fraction between consecutive time steps is then introduced into Equation (16) as a source term, allowing the model to capture the latent heat effects during the melting and solidification processes.

### 3.4. Boundary Conditions

It is well known that the implementation of boundary conditions (BCs) within the lattice Boltzmann method (LBM) is not straightforward. Indeed, several studies have devoted considerable effort to the proper formulation and application of BCs in LBM frameworks [29]. In the present methodology, the boundary conditions are imposed by enforcing the conservation of energy flux at the corresponding boundaries of the computational domain. To accurately represent the physical conditions of the system under study, both Dirichlet and Neumann boundary conditions are employed.

The Dirichlet boundary condition is applied to the left, right, and bottom boundaries of the rectangular domain, where these surfaces are assumed to be maintained at a prescribed temperature $T_{wall}$ during the laser melting process. In order to enforce this condition, the unknown particle distribution functions at these boundaries must be appropriately evaluated. This requirement arises because, during the streaming step of the LBM algorithm, not all distribution functions can be directly obtained.

From the two-dimensional scheme shown in Figure 5, it can be observed that the distribution functions $f_{2}$, $f_{6}$, and $f_{9}$ at the left boundary, $f_{4}$, $f_{7}$, and $f_{8}$ at the right boundary, and $f_{3}$, $f_{6}$, and $f_{7}$ at the bottom boundary remain unknown, since these groups of distribution functions propagate toward the right, left, and top directions, respectively. To address this issue, three groups of distribution functions are defined based on the flux conservation principle, as described below:

(19) $$\left\{\begin{array}{c} f_{2}\left(0,j\right)+f_{4}\left(0,j\right)=w_{2}T_{wall}+w_{4}T_{wall}\\ f_{6}\left(0,j\right)+f_{8}\left(0,j\right)=w_{6}T_{wall}+w_{8}T_{wall}\\ f_{9}\left(0,j\right)+f_{7}\left(0,j\right)=w_{9}T_{wall}+w_{7}T_{wall} \end{array}\right.$$

(20) $$\left\{\begin{array}{c} f_{4}\left(L,j\right)+f_{2}\left(L,j\right)=w_{4}T_{wall}+w_{2}T_{wall}\\ f_{7}\left(L,j\right)+f_{9}\left(L,j\right)=w_{7}T_{wall}+w_{9}T_{wall}\\ f_{8}\left(L,j\right)+f_{6}\left(L,j\right)=w_{8}T_{wall}+w_{6}T_{wall} \end{array}\right.$$

(21) $$\left\{\begin{array}{c} f_{3}\left(i,0\right)+f_{5}\left(i,0\right)=w_{3}T_{wall}+w_{5}T_{wall}\\ f_{6}\left(i,0\right)+f_{8}\left(i,0\right)=w_{6}T_{wall}+w_{8}T_{wall}\\ f_{7}\left(i,0\right)+f_{9}\left(i,0\right)=w_{7}T_{wall}+w_{9}T_{wall} \end{array}\right.$$

Equations (19)–(21) are defined by doing a detailed flux balance at the boundary x=0, x=Lx, and y=0. For instance, at x=0 it is rational to write that $f_{2}^{eq}-f_{2}+f_{4}^{eq}-f_{4}=0$, where $f_{2}^{eq}=w_{2}T_{wall}$ and $f_{4}^{eq}=w_{4}T_{wall}$; as a result $f_{2}$ can be calculated. In this way, applying the flux conservation equation for the other distribution functions at the left, right, and bottom limits, it is possible to calculate the unknown distribution functions. It is worth mentioning that there is another way to analyze the BCs considering a residual temperature. The details can be found in [27], although both methodologies are in good agreement on this aspect.

The Neumann boundary condition is used to describe the heat flux and convective heat transfer at the top side of the simulation domain, i.e., at y=Ly, where there exists the convective heat transfer zone, as well as the laser contact zone. Thus, applying the energy conservation equation for unsteady-state heat transfer, the thermal boundary condition can be established as follows:

(22) $$-\;k\frac{\partial T}{\partial N}+w_{k}\dot{q}A+h\left(T-T_{a}\right)\;\Delta x=\rho C_{p}V_{0}\frac{\partial T}{\partial t}$$

where *N* denotes the normal vector of the surface at which the heat fluxes are imposed. The parameters *k*, ρ, and $C_{p}$ represent the thermal conductivity, density, and specific heat capacity of the medium, respectively. The term $q^{\prime \prime }$ corresponds to the heat flux applied at the laser–material interaction zone, i.e., the laser heat flux described by a Gaussian spatial distribution as adopted in [10]. The convective heat transfer coefficient *h* accounts for heat exchange between the metallic powder bed and the surrounding environment and is assumed to be constant. Finally, $V_{0}=\Delta x\;\Delta y$ represents the volume of a single lattice cell.

The important question is how to relate Equation (22) with the unknown distribution functions attached at the heat flux and convective heat zone of the rectangular system. For instance, the relation for the convective heat transfer coefficient is given as:

(23) $$-k\frac{\partial T}{\partial N}+h\left(T-T_{a}\right)\;\Delta x=\rho C_{p}V_{0}\frac{\partial T}{\partial t}$$

where ∂T/∂N describes the heat conduction term in the *j*-direction between the corresponding nodes (i,n) and (i,n−1). Then, the temperature gradient can be related as −kΔx[(T(i,n−1)−T(i,n))/Δy], which is a well-known term by FDM. Substituting into Equation (23) gives

(24) $$-k\Delta x\left[\frac{T(i,n-1)-T(i,n)}{\Delta y}\right]-h\Delta x\left(T\left(i,n\right)-T_{a}\right)=\rho C_{p}V_{0}\frac{T^{t+\Delta t}\left(i,n\right)-T\left(i,n\right)}{\Delta t}$$

where the corresponding values for T(i,n), T(i,n−1), and $T^{t+\Delta t}\left(i,n\right)$ in the LB formulation are assumed as follows:

(25) $$\begin{array}{cc} T(i,n) & =w_{3}\;f_{3}\left(i,n\right)+w_{5}\;f_{5}\left(i,n\right) \end{array}$$

(26) $$\begin{array}{cc} T(i,n-1) & =w_{3}\;f_{3}\left(i,n-1\right)+w_{5}\;f_{5}\left(i,n-1\right) \end{array}$$

(27) $$\begin{array}{cc} T^{t+\Delta t}\left(i,n\right) & =w_{3}\;f_{3}\left(i,n\right)+w_{5}\;f_{5}\left(i,n\right) \end{array}$$

Note that the distribution functions $f_{3}$ and $f_{5}$ in Equation (27) correspond to the time level t+Δt, where the distribution function $f_{3}$ is obtained from the streaming process and $f_{5}$ is the distribution function that needs to be determined. The distribution functions $f_{3}$ and $f_{5}$ in Equations (25) and (26) are known from the previous time step *t*. Therefore, by substituting Equations (25)–(27) into Equation (24) and solving for the unknown distribution function $f_{5}$ at the new time step, the resultant equation will be:

(28) $$\begin{array}{c} f_{5}\left(i,n\right)=\frac{\alpha \Delta t}{{\left(\Delta x\right)}^{2}}\left[f_{3}\left(i,n-1\right)+f_{5}\left(i,n-1\right)\right]+\left(1-\frac{\alpha \Delta t}{{\left(\Delta x\right)}^{2}}\right)\left[f_{3}\left(i,n\right)+f_{5}\left(i,n\right)\right]-f_{3}\left(i,n\right)\\ +\frac{\alpha \Delta t}{{\left(\Delta x\right)}^{2}}\left(\frac{h\;\Delta x}{k}\right)\left[w_{3}T_{a}+w_{5}T_{a}\right] \end{array}$$

where α is the thermal diffusivity of the real system, defined as $\alpha =k/(\rho C_{p})$. In this way, the unknown distribution function $f_{5}$ at the new time level is related to the energy conservation equation applied in the convective contact zone, as $f_{5}$ cannot be obtained by the streaming process. Similarly, the energy conservation equation for unsteady-state heat transfer is applied for the laser contact zone in order to obtain the unknown distribution function $f_{5}$.

## 4. Results

### 4.1. Microstructure Analysis

**Figure 6:**
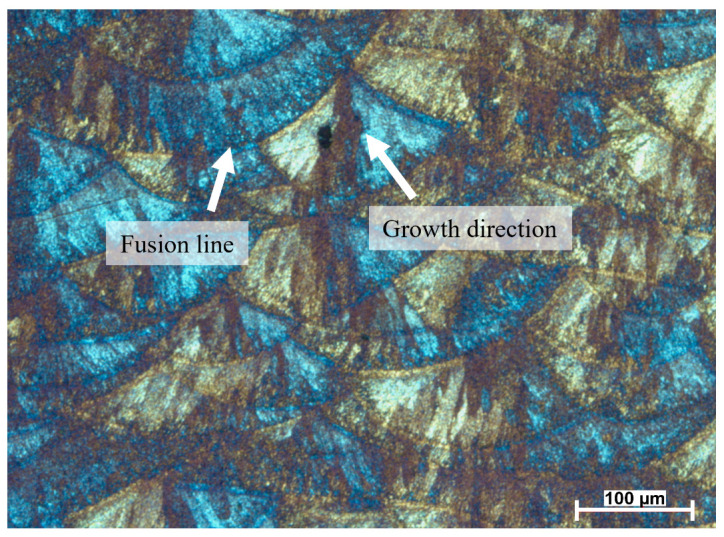
Optical micrograph of the SLM AlSi10Mg microstructure etched with Barker’s reagent and observed under polarized light [18].

Figure 6 shows the microstructure of the AlSi10Mg alloy, where grain growth is directed toward the center of the melt pool. This indicates the formation of columnar grains as a result of directional solidification during the laser melting process. This observation highlights the strong thermal influence of the laser on the material. According to the literature, understanding the melt pool dimensions in SLM materials is essential for predicting the final mechanical properties. In particular, excessive melt pool penetration can lead to undesired phenomena such as entrapped gases at the bottom of the melt pool. These gases may pose serious problems, especially when they are unable to escape due to the rapid solidification inherent to the SLM [30]. The color contrast observed in the optical micrographs is associated with the metallographic preparation and the use of polarized light during the observation of the etched samples. In particular, Barker’s reagent combined with polarized light produces interference colors that reveal grain orientation and solidification features in aluminum alloys. These color variations therefore correspond to crystallographic contrast rather than artificial image processing.

**Figure 7:**
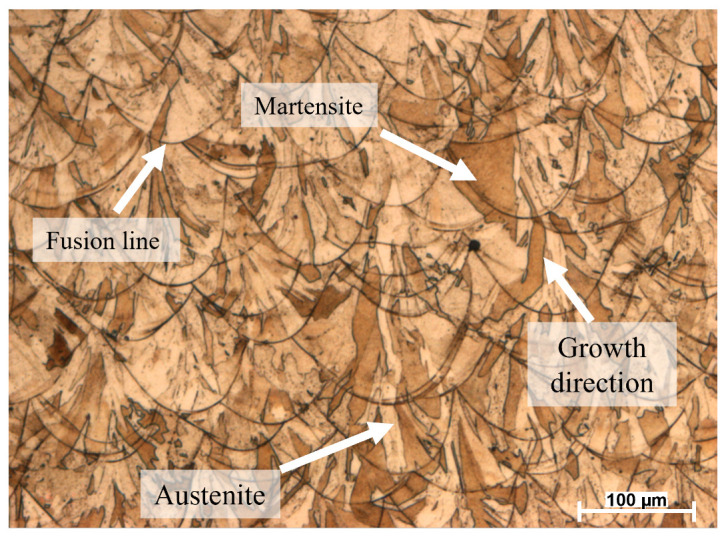
Optical micrograph showing the SLM 316L SS microstructure [18].

On the other hand, the microstructure of SLM-processed 316L SS reveals predominantly columnar grains oriented along the build direction, indicative of directional solidification under high thermal gradients. As shown in Figure 7, the grains grow from the fusion line to the center of the melt pool, i.e., across hundreds or thousands of layers [31]. This is because of the thermal gradients produced by the laser melting process, which are higher at the melt pool center than along the fusion line. The grain structure is characterized by martensite, appearing as a dark brown tone, and austenite, which is observed as a lighter brown phase. Nevertheless, a gas-induced spherical defect seems to be present in the microstructure. According to Xiong et al. [32], the circular shape of that defect allows us to deduce that it could formed by a gas induced from the liquid metal during the rapid solidification process.

### 4.2. Experimental Melt Pool Dimensions

**Figure 8:**
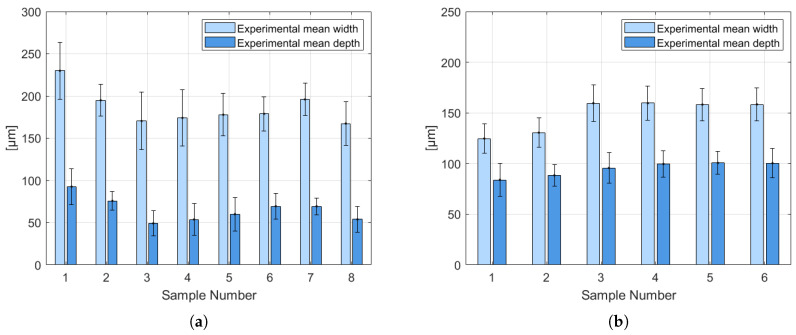
Experimental melt pool dimensions obtained by optical microscopy: (**a**) AlSi10Mg alloy and (**b**) 316L SS.

Figure 8 presents the experimental measurements of melt pool width and depth obtained from SLM-processed AlSi10Mg and 316L SS samples. The reported values correspond to the average melt pool dimensions measured for each sample. For the AlSi10Mg alloy (Figure 8a), the melt pool width and depth were evaluated across eight different samples. Samples 1 to 4 correspond to measurements taken in the XZ plane, whereas samples 5 to 8 correspond to the YZ plane. In the XZ plane, an average of 80 melt pool measurements per sample was recorded for both width and depth, while in the YZ plane, an average of 91 melt pool measurements per sample was obtained.

The experimental results show a noticeable variation in melt pool geometry among the samples. The largest melt pool width is observed for sample 1, with a value of approximately 230 μm, whereas the smallest width corresponds to sample 8, with an average value close to 168 μm. Regarding melt pool depth, sample 1 also exhibits the highest value, approximately 93 μm, while the minimum depth is registered for sample 3, with an average depth close to 50 μm. These variations can be associated with local thermal conditions during laser scanning, such as energy input fluctuations and melt pool stability.

In the case of the 316L SS samples (Figure 8b), six specimens were analyzed. For samples 1 to 3, corresponding to measurements in the XZ plane, an average of 80 melt pool width and depth measurements per sample was recorded. For samples 4 to 6, corresponding to the XY plane, the average number of melt pool measurements per sample was 51. As observed in Figure 8b, the smallest melt pool width and depth are obtained for sample 1, with approximate values of 125 μm and 84 μm, respectively. The maximum melt pool width is registered for sample 4, reaching values close to 160 μm, whereas the largest melt pool depth is observed for sample 5, with an average value of approximately 101 μm. Compared to the AlSi10Mg alloy, the 316L SS samples exhibit deeper melt pools for similar width ranges, which can be attributed to differences in thermophysical properties, particularly thermal conductivity and laser energy absorptivity.

The melt pool dimensions obtained in this study are relatively similar to those reported by Kamath et al. [33], who investigated single-track experiments on 316L SS using different sets of processing parameters. In their work, the laser power was varied from 150 W to 400 W, while the scanning speed ranged from 500 mm/s to 1800 mm/s. The smallest melt pool width and depth reported were approximately 79 μm and 30 μm, respectively. In contrast, the largest melt pool width reached about 123 μm for a laser power of 200 W and a scanning speed of 800 mm/s, whereas the maximum melt pool depth of approximately 195 μm was obtained using a laser power of 200 W and a scanning speed of 500 mm/s. These melt pool dimensions were obtained under single-track processing conditions.

Overall, the experimental results confirm that melt pool geometry is highly sensitive to processing conditions and material properties. These measurements provide a solid experimental basis for the validation of the lattice Boltzmann thermal model developed in this work, particularly in terms of predicting melt pool width and depth under selective laser melting conditions.

### 4.3. Grid Refinement and Temporal Sensitivity Analysis

Before comparing the numerical predictions with the experimental observations, a grid refinement analysis was carried out in order to evaluate the influence of spatial discretization on the predicted melt pool geometry and temperature field. The analysis was performed by considering a representative configuration that consisted of a single deposited layer and a single laser spot impacting the top surface of the computational domain. The horizontal domain length was kept identical to that used in the full simulations, while the vertical domain height was reduced to represent one deposited layer over the substrate.

Four different grid resolutions were considered, 100×53, 200×106, 500×265, and 1000×530 lattice cells, corresponding to progressively refined discretizations. For each grid resolution, the melt pool width, melt pool depth, and maximum temperature at the center of the laser spot were monitored.

In the present lattice Boltzmann formulation, normalized lattice units are adopted, where Δx=Δt=1. Consequently, the physical spatial and temporal resolutions vary with the selected grid. As the number of lattice nodes increases, the physical cell size decreases and the number of time steps required to represent the physical time interval increases accordingly. Therefore, the present study represents a coupled spatial–temporal sensitivity analysis consistent with the LBM formulation.

Table 4 summarizes the predicted melt pool width, melt pool depth, and maximum temperature for each grid resolution together with the relative deviation with respect to the finest mesh (1000×530). The results show that the predicted melt pool characteristics progressively stabilize as the mesh is refined. Although fully asymptotic convergence is not reached within the tested discretizations, the variations between the intermediate and finer meshes become significantly smaller, indicating that the main thermal trends and melt pool geometry are adequately captured.

Further evidence of numerical stabilization can be observed by comparing the two finest meshes. The variation between the 500×265 and 1000×530 grids is approximately 6.2% for melt pool width, 5.3% for melt pool depth, and 2.2% for the maximum temperature. These relatively small differences indicate that the predicted melt pool characteristics approach a stable numerical solution as the spatial resolution increases.

Considering the balance between computational cost and numerical accuracy, the grid resolution of 200×112 cells (used in the full simulations with two deposited layers) was adopted for the remainder of this study. This discretization provides a reasonable compromise between computational efficiency and predictive capability.

**Figure 9:**
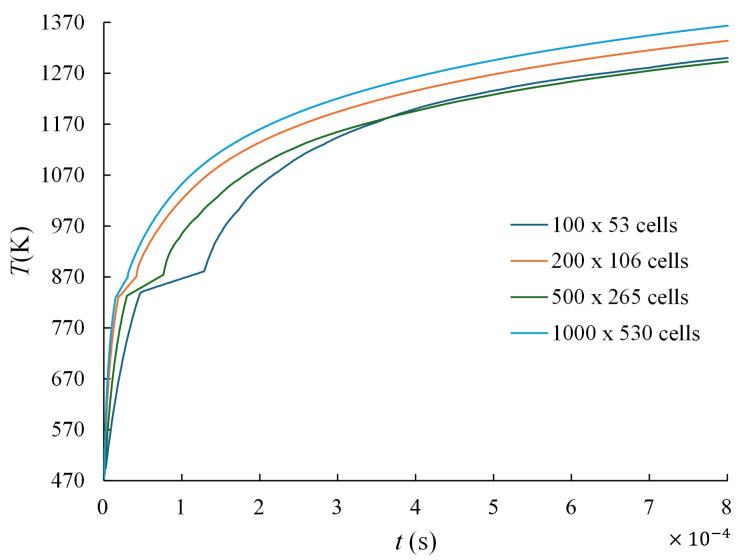
Temporal evolution of the maximum temperature at the laser spot for different grid resolutions used in the grid refinement study performed for the AlSi10Mg alloy.

In addition to the spatial refinement analysis, the temporal evolution of the maximum temperature at the laser spot was also examined for the different grid resolutions. Figure 9 presents the temperature evolution curves obtained for each mesh. As the spatial resolution increases, the predicted temperature field exhibits a progressively smoother and more stable evolution. The similarity of the temperature trends among the intermediate and finer meshes further confirms that the selected discretization captures the dominant thermal behavior of the process.

### 4.4. Experimental vs. LBM Melt Pool Comparison

**Figure 10:**
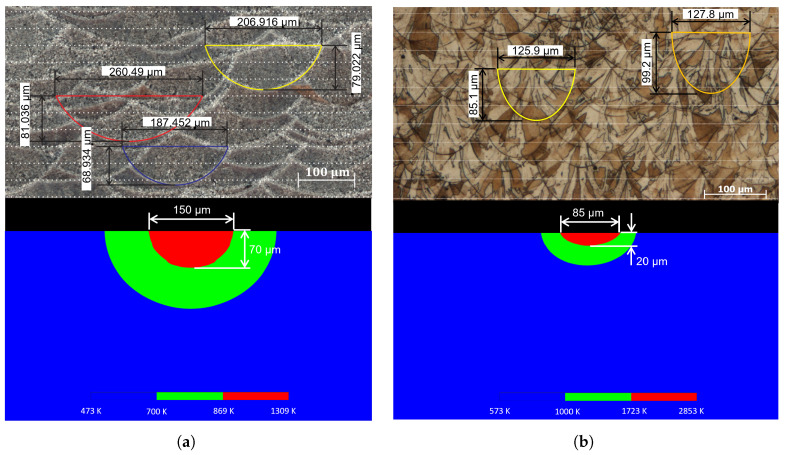
Comparison between experimental [18] and numerical melt pool geometry obtained by LBM: (**a**) AlSi10Mg alloy and (**b**) 316L SS.

Figure 10 presents a direct comparison between the experimentally measured melt pool geometry and the corresponding numerical results obtained using the lattice Boltzmann method (LBM) for both AlSi10Mg and 316L SS processed by selective laser melting.

For the AlSi10Mg alloy (Figure 10a), the experimental measurements reveal melt pool widths of approximately 206 μm, 260 μm, and 187 μm, while the corresponding melt pool depths are about 79 μm, 81 μm, and 68 μm. These values indicate that the laser penetrates beyond the nominal powder layer thickness of 30 μm, reaching depths equivalent to slightly more than two deposited layers. Moreover, it is well established that the melt pool geometry, particularly its width and depth, is strongly dependent on the applied laser power [34].

The numerical results obtained from the LBM simulation predict a melt pool width of approximately 150 μm and a depth close to 70 μm. From a physical standpoint, these results indicate that the laser fully melts the current powder layer and induces a re-melting phenomenon in the previously consolidated material. The predicted penetration depth corresponds to more than two layer thicknesses, which is consistent with the experimental observations.

For the 316L SS alloy (Figure 10b), the experimental measurements show melt pool widths of approximately 138 μm and 158 μm, with corresponding depths of about 89 μm and 100 μm. Considering a powder layer thickness of 30 μm, the maximum experimental depth indicates that the laser penetrates up to the equivalent of nearly three layers, suggesting a stronger interaction between the laser and the material.

In contrast, the numerical LBM results predict a melt pool width of approximately 85 μm and a significantly lower depth of about 20 μm. These results indicate that, for this material, the current numerical model underestimates the melt pool penetration depth and does not fully reproduce the experimental thermal behavior. The reduced numerical penetration suggests that additional material-dependent effects, such as differences in absorptivity, thermal conductivity, phase-change dynamics, or melt pool convection, may need to be incorporated or refined in the model to improve its predictive capability for 316L SS.

The comparison presented in Figure 10 highlights the predictive capability of the proposed LBM-based thermal model. For the AlSi10Mg alloy, the numerical results reproduce the characteristic semi-elliptical morphology of the melt pool as well as its penetration behavior into the substrate. Although some deviations in melt pool dimensions are observed, the predicted geometry remains within the same order of magnitude as the experimental measurements.

In the case of the SLM 316L SS alloy (Figure 10b), the numerical results capture the general morphology of the melt pool but show larger discrepancies in depth. These differences can be attributed to the higher sensitivity of 316L SS to thermophysical properties, melt flow effects, and laser absorption conditions. Nevertheless, the numerical model successfully reproduces the main thermal features governing melt pool formation. These results demonstrate that the proposed LBM formulation provides a reliable framework for predicting melt pool geometry and offers a useful computational tool for preliminary analysis and optimization of SLM processing parameters.

Table 5 presents the quantitative comparison between the melt pool dimensions obtained experimentally and those predicted by the LBM simulations for both AlSi10Mg and 316L SS alloys. For this analysis, average melt pool width and depth values were calculated from measurements performed in the XZ and YZ planes and subsequently compared with the numerical predictions obtained from the LBM model.

The quantitative comparison shows that the LBM model predicts the melt pool geometry of the AlSi10Mg alloy with reasonable agreement. In particular, the relative error remains below 20% for the melt pool width and below 10% for the melt pool depth, indicating that the proposed thermal model captures the dominant heat transfer mechanisms governing melt pool formation for this alloy.

It is important to highlight that the volumetric energy density employed to process the SLM AlSi10Mg samples in this study was 333.33 J/mm^3^. By comparison, Li et al. [11] reported that an energy density of 500 J/mm^3^ was required to achieve a melt pool width and depth of 111.4 μm and 67.5 μm, respectively. In that study, it was also confirmed that deposited layers with a thickness of 50 μm were well connected with the calculated penetration depth.

Therefore, for the AlSi10Mg alloy analyzed in the present work, the LBM-based thermal model demonstrates good qualitative and quantitative agreement with the experimentally observed melt pool geometry.

In contrast, larger discrepancies are observed for the 316L SS alloy, where the numerical model underestimates both melt pool width and depth. The relative errors reach 42.75% for width and 78.89% for depth. These differences suggest that additional physical phenomena, such as melt pool convection, evaporation effects, and recoil pressure induced by metal vaporization, may play a more significant role in the thermal behavior of 316L SS during the SLM process and are not fully represented in the simplified thermal formulation adopted in the present study.

The larger deviation observed for the 316L SS alloy can be better understood by comparing its thermo-physical properties with those of AlSi10Mg. In the present study, 316L SS exhibits a higher laser absorptivity (A=0.35) than AlSi10Mg (A=0.09), but also a much higher melting range, with solidus and liquidus temperatures of 1658 K and 1723 K, respectively, compared with 830 K and 869 K for AlSi10Mg. In addition, the thermal conductivity of 316L SS at 573 K is considerably lower (k=18 W/m·K) than that of AlSi10Mg at 473 K (k=159 W/m·K), while its density is substantially higher (7980 kg/m^3^ for 316L SS versus 2650 kg/m^3^ for AlSi10Mg). These differences indicate that the thermal response of 316L SS during SLM is governed by a more complex balance between laser absorption, heat accumulation, and melting resistance. Consequently, the simplified thermal model adopted in this work is able to capture the melt pool behavior of AlSi10Mg with better agreement, whereas for 316L SS, the omission of additional physical effects, such as melt pool convection and evaporation-induced recoil pressure, may have a stronger influence on the predicted melt pool geometry.

Overall, the comparison highlights that while the proposed LBM framework provides a reliable prediction of melt pool geometry for AlSi10Mg, further model calibration and material-specific adjustments are required to achieve a similar level of agreement for 316L SS under selective laser melting conditions.

### 4.5. Thermal Effect Predicted by LBM: Temperature Profiles Across Layers and Laser Spots

To further evaluate the thermal response predicted by the LBM-based SLM model, vertical temperature profiles were extracted along the *y*-axis of the computational domain, passing through the center of each laser spot. For each deposited layer, three spot positions were simulated, and the corresponding temperature distributions were recorded after the laser impact.

**Figure 11:**
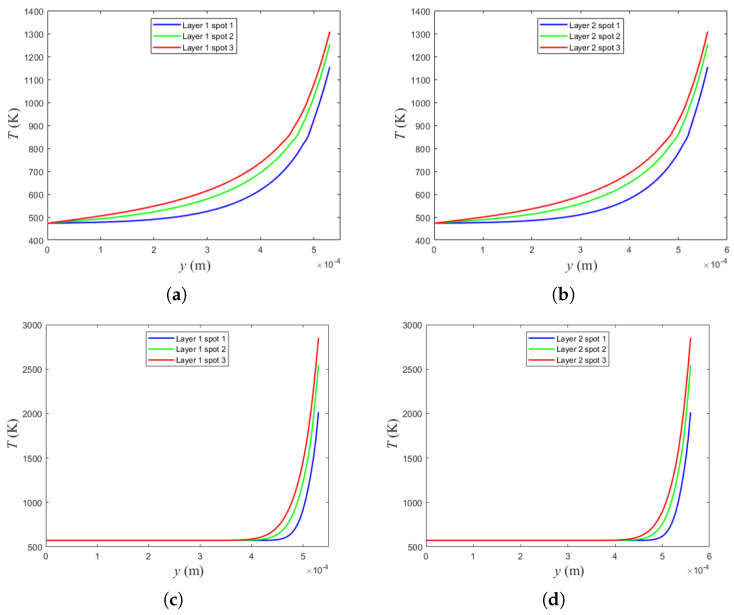
Temperature profiles predicted by the LBM model along the vertical *y*-direction through the spot centers: (**a**) SLM AlSi10Mg, layer 1 (three spots); (**b**) SLM AlSi10Mg, layer 2 (three spots); (**c**) SLM 316L SS, layer 1 (three spots); and (**d**) SLM 316L SS, layer 2 (three spots).

Figure 11 summarizes these results. Figure 11a,b correspond to the AlSi10Mg alloy for the first and second deposited layers, respectively. In both layers, the thermal response increases from spot 1 to spot 3, while remaining nearly unchanged between consecutive layers. The maximum temperatures for the AlSi10Mg case are approximately 1155 K, 1254 K, and 1308 K for spot 1, spot 2, and spot 3, respectively. This indicates that the model is able to represent both the laser scanning sequence (spot-to-spot evolution) and the effect of sequential material deposition (layer-to-layer evolution) without introducing artificial thermal drift between layers under the same process conditions.

A similar analysis was performed for the 316L SS, as shown in Figure 11c,d for the first and second layers, respectively. In this case, the predicted maximum temperatures are significantly higher, reaching approximately 2014 K, 2546 K, and 2853 K for spot 1, spot 2, and spot 3. As in the AlSi10Mg case, the profiles remain consistent between layer 1 and layer 2, supporting the capability of the LBM framework to track the laser movement and the sequential deposition of material while preserving a coherent thermal response across layers.

### 4.6. LM–MS Interfacial Evolution During Laser Scanning

**Figure 12:**
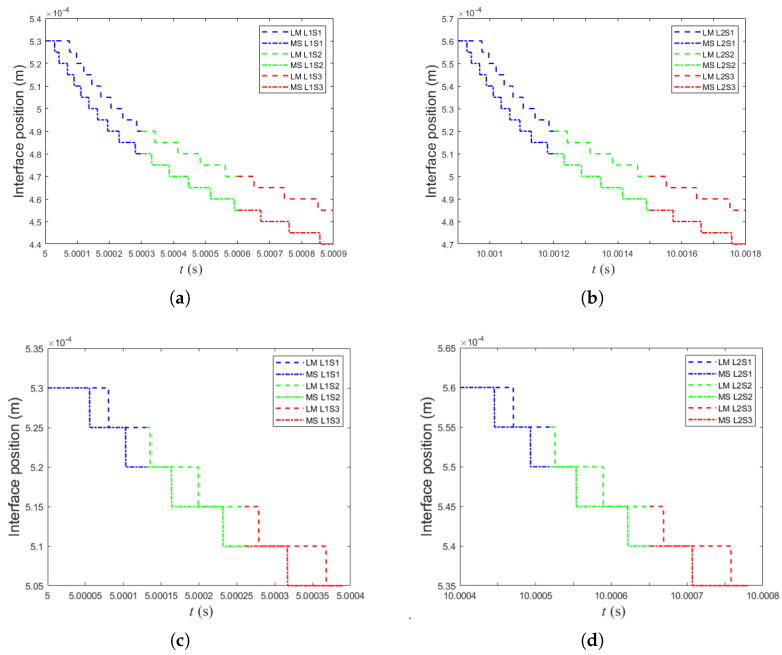
LBM-predicted liquid/mushy (LM) and mushy/solid (MS) interfacial positions for three laser spots: (**a**) AlSi10Mg layer 1, (**b**) AlSi10Mg layer 2, (**c**) 316L SS layer 1, and (**d**) 316L SS layer 2. The region between LM and MS curves represents the extent of the mushy zone.

Figure 12 presents the evolution of the liquid/mushy (LM) and mushy/solid (MS) interfacial positions predicted by the LBM model during laser scanning. These curves track the penetration depth (interface position along *y*) of the isotherms associated with the liquidus and solidus limits, respectively. Therefore, the region between the LM and MS curves represents the extent of the mushy zone, i.e., the partially melted region that develops under transient heating and solidification conditions.

For the AlSi10Mg alloy (Figure 12a,b), the LM and MS interfaces move deeper as the laser advances from spot 1 to spot 3, indicating an increased melt penetration and a progressive growth of the mushy region. This trend is consistent with the cumulative thermal effect produced by successive laser impacts, where heat diffusion and partial remelting contribute to an expanded LM–MS separation. In addition, when comparing layer 1 and layer 2, the interfacial trajectories remain comparable, suggesting that the model captures both the laser motion and the layer-by-layer deposition while preserving similar thermal gradients under the selected processing conditions.

In contrast, for the 316L SS (Figure 12c,d), the LM and MS interfacial positions tend to decrease more rapidly after each laser impact, and the separation between both interfaces is less pronounced. This behavior indicates a thinner and shorter-lived mushy region, which can be attributed to a sharper thermal decay and faster solidification response within the simulated domain. Such a response is consistent with the steeper temperature profiles previously observed for 316L SS, where the thermal field relaxes more quickly away from the laser interaction zone, limiting the spatial and temporal persistence of partially melted material.

## 5. Discussion

The results obtained in this work provide a comprehensive assessment of the thermal behavior and melt pool characteristics during the selective laser melting (SLM) process through a combined experimental and numerical approach. Although the real powder bed is inherently heterogeneous, the present work adopts a homogeneous effective-medium assumption, which has been shown to provide reliable predictions of melt pool geometry and thermal behavior. The lattice Boltzmann method (LBM) implemented in this study demonstrates the capability to capture key thermal phenomena associated with laser–material interaction, phase change, and layer-by-layer deposition. Comparable physical mechanisms in SLM were analyzed in [34] using two homogeneous modeling approaches to address the melting and solidification behavior of the powder bed.

Experimental microstructural observations for both AlSi10Mg and 316L SS revealed predominantly columnar grain structures aligned with the build direction, confirming the presence of strong thermal gradients and directional solidification during SLM. These observations are consistent with the predicted thermal fields obtained from the LBM simulations, where steep temperature gradients develop near the laser impact zone and decay rapidly away from the melt pool.

Quantitative comparison between experimental and numerical melt pool dimensions showed good agreement for the AlSi10Mg alloy. The LBM model successfully predicted melt pool widths and depths on the order of two to three powder layer thicknesses, capturing the remelting phenomenon commonly observed in SLM processes. This agreement suggests that the thermal formulation, boundary conditions, and phase-change implementation are adequate for aluminum-based alloys under the selected processing conditions.

In contrast, the comparison for 316L SS revealed noticeable discrepancies, particularly in the predicted melt pool depth. While the experimental results indicated deeper melt pools with penetration reaching up to three layer thicknesses, the numerical model underestimated the melt pool depth. This behavior is attributed to the higher thermal gradients, faster heat dissipation, and complex thermophysical behavior of 316L SS, which may require further refinement of material properties, laser absorption modeling, and boundary condition treatment. In fact, recoil pressure induced by metal evaporation has been identified as a critical factor influencing melt pool dynamics and geometry during the SLM process of 316L stainless steel [35]. Incorporating such effects could further improve the predictive capability of the present thermal model.

The analysis of temperature profiles across multiple laser spots and deposited layers demonstrated that the LBM framework can effectively model laser scanning strategies and successive layer deposition. For both materials, the peak temperatures increased with consecutive laser spots, while remaining relatively consistent between layers, highlighting the thermal stability of the process under fixed parameters.

Additionally, the evolution of liquid–mushy (LM) and mushy–solid (MS) interfacial positions provided further insight into solidification dynamics. For AlSi10Mg, a well-defined mushy region was observed, indicating sustained thermal gradients and extended solidification intervals. Conversely, the 316L SS alloy exhibited narrower mushy regions, consistent with the sharper temperature gradients and faster cooling rates identified in the temperature profile analysis.

Overall, the results indicate that while the current LBM-based thermal model is robust for predicting thermal behavior and melt pool geometry in AlSi10Mg, additional model calibration is necessary for alloys with higher melting temperatures and thermal conductivity variations, such as 316L SS.

## 6. Conclusions

In this study, a two-dimensional thermal model based on the lattice Boltzmann method (LBM) was developed to simulate the selective laser melting (SLM) process of AlSi10Mg and 316L stainless steel (316L SS) alloys. The proposed model accounts for laser–material interaction, transient heat transfer, phase change phenomena, and layer-by-layer material deposition, enabling a detailed description of the thermal mechanisms governing melt pool formation.

A mesh refinement study was performed to evaluate the numerical stability and grid independence of the model. Four mesh resolutions ranging from 100×53 to 1000×530 cells were analyzed. The results showed that the predicted melt pool dimensions converge as the mesh resolution increases, with variations below approximately 7% between the selected computational mesh (200×106 cells) and the finest grid considered. This confirms that the adopted discretization provides a suitable balance between numerical accuracy and computational efficiency.

Experimental characterization of melt pool geometry was employed to validate the numerical predictions. For the AlSi10Mg alloy, the LBM model showed good quantitative agreement with the experimental measurements. The predicted melt pool width and depth were approximately 150 μm and 70 μm, respectively, corresponding to relative errors of 19.13% in width and 7.58% in depth compared with the averaged experimental values. From a physical standpoint, the numerical results correctly capture the full melting of the current powder layer and the remelting of previously consolidated material, with penetration depths exceeding two layer thicknesses. These results confirm the capability of the proposed LBM-based thermal model to reliably reproduce melt pool geometry and remelting behavior for AlSi10Mg processed by SLM.

For the 316L SS alloy, the numerical results exhibited qualitative agreement with the experimentally observed melt pool morphology; however, the model underestimated the melt pool dimensions. The numerical predictions yielded a melt pool width of approximately 85 μm and a depth close to 20 μm, resulting in relative errors of 42.75% and 78.89%, respectively. These differences highlight the strong sensitivity of the SLM process to material thermophysical properties, such as thermal conductivity, absorptivity, and melting temperature range.

Overall, the results demonstrate that the proposed LBM framework provides a computationally efficient approach for predicting melt pool geometry and thermal fields during the SLM process, particularly for alloys with moderate melting temperatures such as AlSi10Mg. The model captures the dominant heat transfer mechanisms governing melt pool formation while maintaining relatively low computational cost.

Future work will focus on extending the present formulation by incorporating additional physical phenomena, including melt pool convection driven by Marangoni effects, recoil pressure induced by metal evaporation, and temperature-dependent thermophysical properties. These improvements are expected to enhance the predictive capability of the model, particularly for high-melting-point alloys such as 316L stainless steel.

## Figures and Tables

**Table 2:** Elemental composition (wt. %) of the 316L SS metallic powder and SLM sample [18].

Element	Cr	Cu	Mn	Mo	Ni	Si	Fe
Powder	18.38	–	1.61	2.48	11.65	1.00	Bal.
SLM sample	18.67	0.04	1.13	2.55	11.84	–	Bal.

**Table 3:** SLM processing parameters and thermo-physical properties of the AlSi10Mg and 316L stainless steel alloys [11,18,22,23,24,25,26].

Parameter	AlSi10Mg	316L SS
Ambient temperature, $T_{a}$	298 K	298 K
Layer thickness, Lt	30 μm	30 μm
Laser diameter, *D*	70 μm	70 μm
Laser power, *P*	100 W	400 W
Hatch spacing, HS	100 μm	110 μm
Scanning speed, *v*	100 mm/s	230 mm/s
Preheating temperature, $T_{0}$	473 K	573 K
Density (solid), ρ	2650 kg/m^3^	7980 kg/m^3^
Thermal conductivity (solid), *k*	159 W/m·K	18 W/m·K
Specific heat capacity (solid), $C_{p}$	797 J/kg·K	545 J/kg·K
Solidus temperature, $T_{s}$	830 K	1658 K
Liquidus temperature, $T_{l}$	869 K	1723 K
Latent heat of phase change, *L*	423 kJ/kg	286 kJ/kg
Convection heat transfer coefficient, *h*	20 W/m^2^·K	20 W/m^2^·K
Absorptivity, *A*	0.09	0.35

**Table 4:** Grid refinement study showing melt pool dimensions and maximum temperature together with the relative deviation with respect to the finest mesh.

Grid (Cells)	Width [μm]	Error (%)	Depth [μm]	Error (%)	$T_{\max}$ [K]	Error (%)
100×53	120	25.9	70	7.9	1300.1	4.7
200×106	140	13.6	65	14.5	1293.1	5.2
500×265	152	6.2	72	5.3	1333.9	2.2
1000×530	162	0.0	76	0.0	1363.5	0.0

**Table 5:** Quantitative comparison between experimental melt pool dimensions and LBM numerical predictions for the alloys analyzed in this study.

Alloy	Parameter	Plane XZ	Plane YZ	Average	LBM Simulation	Error (%)
AlSi10Mg	Width (μm)	192.28	178.67	185.48	150	19.13
	Depth (μm)	67.88	62.25	65.06	70	7.58
316L SS	Width (μm)	138.24	158.71	148.47	85	42.75
	Depth (μm)	89.30	100.17	94.73	20	78.89

## Data Availability

The original contributions presented in this study are included in the article. Further inquiries can be directed to the corresponding author.
